# Smart Microbiomes: How AI Is Revolutionizing Personalized Medicine

**DOI:** 10.3390/bioengineering12090944

**Published:** 2025-08-31

**Authors:** Luana Alexandrescu, Ionut Tiberiu Tofolean, Laura Maria Condur, Doina Ecaterina Tofolean, Alina Doina Nicoara, Lucian Serbanescu, Elena Rusu, Andreea Nelson Twakor, Eugen Dumitru, Andrei Dumitru, Cristina Tocia, Lucian Flavius Herlo, Daria Maria Alexandrescu, Alina Mihaela Stanigut

**Affiliations:** 1 Gastroenterology Department, “Sf. Apostol Andrei” Emergency County Hospital, 145 Tomis Blvd., 900591 Constanta, Romania; alexandrescu_l@yahoo.com (L.A.); eugen.dumitru@yahoo.com (E.D.); dr.andreidumitru@gmail.com (A.D.); cristina.tocia@yahoo.com (C.T.); 2 Medicine Faculty, “Ovidius” University of Constanta, 1 Universitatii Street, 900470 Constanta, Romania; lauracondur@yahoo.com (L.M.C.); tofoleandoina@yahoo.com (D.E.T.); lucian.serbanescu@365.univ-ovidius.ro (L.S.); alina.stanigut@365.univ-ovidius.ro (A.M.S.); 3 Pneumology Department, “Sf. Apostol Andrei” Emergency County Hospital, 145 Tomis Blvd., 900591 Constanta, Romania; 4 Internal Medicine Department, “Sf. Apostol Andrei” Emergency County Hospital, 145 Tomis Blvd., 900591 Constanta, Romania; alina.nicoara@365.univ-ovidius.ro (A.D.N.); andreea.purcaru@365.univ-ovidius.ro (A.N.T.); 5 Faculty of Medicine, Titu Maiorescu University, 050044 Bucharest, Romania; alexandrescu_daria@yahoo.com; 6 Doctoral School, Victor Babes University of Medicine and Pharmacy, 300041 Timisoara, Romania; flavius.herlo@umft.ro; 7 Nephrology Department, “Sf. Apostol Andrei” Emergency County Hospital, 145 Tomis Blvd., 900591 Constanta, Romania

**Keywords:** gut microbiota, chronic inflammation, probiotics, gastrointestinal disorders, metabolic diseases, autoimmune diseases, machine learning, artificial intelligence

## Abstract

**Background:** Recent studies have shown that gut microbiota have important roles in different human diseases. There has been an ever-increasing application of high-throughput technologies for the characterization of microbial ecosystems. This led to an explosion of various molecular profiling data, and the analysis of such data has shown that machine-learning algorithms have been useful in identifying key molecular signatures. **Results:** In this review, we first analyze how dysbiosis of the intestinal microbiota relates to human disease and how possible modulation of the gut microbial ecosystem may be used for disease intervention. Further, we introduce categories and the workflows of different machine-learning approaches and how they perform integrative analysis of multi-omics data. Last, we review advances of machine learning in gut microbiome applications and discuss challenges it faces. **Conclusions:** We conclude that machine learning is indeed well suited for analyzing gut microbiome and that these approaches are beneficial for developing gut microbe-targeted therapies, helping in achieving personalized and precision medicine.

## 1. Introduction

The human intestine harbors a vast community of commensal microorganisms, collectively known as the gut microbiota. These microbes, numbering in the trillions, collectively possess a genetic repertoire estimated to be over 100 times larger than that of the human genome [1].

In this review, we define dysbiosis not only as a broad imbalance of the gut microbiota but also in terms of measurable ecological features. Dysbiosis may involve the loss of keystone taxa, a reduction in functional diversity, or a shift in overall community structure. To quantify these changes, researchers frequently rely on ecological diversity and dissimilarity metrics. For example, Bray–Curtis dissimilarity is often used to measure differences in microbial composition between individuals or cohorts, while Shannon and Simpson diversity indices capture both richness and evenness of species distributions within a sample. In addition, evenness metrics help evaluate whether a microbial community is dominated by only a few taxa or is more uniformly distributed. Together, these indices provide a standardized way to capture and compare dysbiotic states across studies.

Prior studies have shown that reduced functional diversity of human gut microbiota is a critical factor in the development of diabetes, obesity, inflammatory bowel disease, liver diseases, and some neurological disorders, including autism spectrum disorder, as well as cardiovascular diseases and cancer of the colon and rectum [2,3,4].

Pathological conditions, which include chronic inflammatory diseases (CIDs), metabolic disorders, and autoimmune diseases are thought to be related to changes in the composition and function of the gut microbiota [5]. CIDs continue to remain a significant health issue due to their low-grade chronic inflammation that is often undiagnosed [6]. Their occurrence has now been associated with the changes that occur in the composition and functions of gut microbiota, which includes inflammatory bowel diseases (IBDs), metabolic syndrome, and autoimmune disorders [7].

Recent advances in artificial intelligence have also introduced hybrid deep-learning architectures and explainable AI methods that can be adapted for microbiome applications [8]. For instance, a hybrid KAN-BiLSTM transformer with a multi-domain dynamic attention model has recently been proposed in the field of cybersecurity [9]. Such architectures, which integrate kernel-based attention networks with bidirectional LSTM layers, hold promise for capturing complex temporal and contextual dependencies in microbiome datasets [10]. In parallel, explainable AI approaches, including SHAP (SHapley Additive exPlanations) and LIME (Local Interpretable Model-agnostic Explanations), provide interpretable outputs that allow researchers and clinicians to better understand the decision-making process of machine learning models [11].

In parallel, there has been an increased interest in the use of probiotics to treat and change the gut microbiota. Restoring microbial homeostasis and lowering inflammatory reactions in the gut is promised by probiotic mixtures with particular selected strains [12].

Several reviews have previously examined the intersection of the gut microbiome and artificial intelligence; however, most have remained descriptive or limited in technical depth [13,14].

Our review differs by integrating a more detailed discussion of methodological considerations such as preprocessing, validation, and bias correction, along with quantitative summaries of landmark AI/ML studies. In addition, we highlight emerging approaches, including autoencoder-based dimensionality reduction, federated learning, and explainable AI. Further, we discuss the concept of dysbiosis and its associations with human diseases. Here, we provide an overview of current microbiome modulation strategies such as diet, probiotics, and fecal microbiota transplantation, and we present the main machine-learning categories and analytical workflows relevant to microbiome data. We also summarize key case studies with quantitative outcomes that illustrate the application of AI/ML in this field; and finally, we examine the challenges, limitations, and future directions that must be addressed to translate these approaches into precision medicine.

### 1.1. The Gut Microbiota in Human Diseases

Numerous investigations have indicated that the disruption of the gut microbiota is believed to be responsible for the development and progression of human diseases, as reported recently. For instance, obesity is linked with higher *Firmicutes/Bacteroidetes* ratios, and more recently, Thingholm et al. argued that obese patients have a different gut microbial profiled with less amounts of *Akkermansia* and *Faecalibacterium* compared to healthy counterparts [15]. Similarly, Deli et al. noted particular changes in gut microbiota composition of prediabetes subjects with lower abundance of *Roseburia hominis* and *Faecalibacterium prausnitziii*, as well as higher concentrations of *Escherichia coli* [16]. Likewise, people suffering from inflammatory bowel disease or Crohn’s disease also have lower microbial diversity or general altered community structure of the gut microbiota, including lesser complex of phylum *Firmicutes* with lower amounts of *Faecalibacterium prausnitzii* and *Bifidobacterium adolescentis* [17]. In colorectal cancer (CRC) metagenomic studies, Yu et al. found that several species like *Parvimonas micra* and *Solobacterium moorei* and 20 microbial gene markers were associated with CRC [18]. Hou et al. documented changes of the gut microbiome in the earliest stages of liver cancer by studying many cross-region cohorts with increased diversity of the phylum *Actinobacteria*, depleted butyrate-producing genera, and enriched lipopolysaccharide-producing genera [19].

### 1.2. The Use of Gut Microbiota as a Potential Treatment Option

There is emerging evidence that suggests the modification of the gut microbiota may be beneficial for disease prevention and/or treatment through dietary interventions, fecal microbiota transplantation (FMT), or probiotic and/or prebiotic supplements [20]. Ghosh et al. found that the Mediterranean diet intervention improves health status and modifies the gut microbiome in older individuals [21]. Several clinical trials have demonstrated the effectiveness of FMT in the treatment of various diseases such as diarrhea, *Clostridioides difficile infection*, and even hepatic steatosis by changing the composition of the intestinal microbial community [22,23]. In a randomized double-blind placebo-controlled trial, Ayesha et al. demonstrated that multi-strain probiotics taken over a period of 6 months profoundly reduced insulin resistance and inflammation in T2D patients [24]. Nachum et al. also showed that the use of probiotic supplements in patients with gestational diabetes had beneficial influence on glycemic control [25]. Additionally, Zhen et al. developed a microbial enzyme inhibitor, which greatly diminished the plasma level of the trimethylamine N-oxide (TMAO) metabolite, which is known to be associated with cardiovascular disease risk [26]. Their study implies that modulation of the harmful gut microbial metabolites production might be a novel intervention approach for disease treatment.

## 2. Methodology

To ensure a comprehensive overview, we conducted a structured literature search in PubMed, Web of Science, and Google Scholar covering the years 2010 to 2025.

The search focused on studies applying artificial intelligence and machine-learning approaches to gut microbiome datasets.

Inclusion criteria were: (i) original research articles, (ii) studies conducted in human cohorts or large-scale animal models, (iii) explicit application of AI/ML algorithms, and (iv) relevance to disease classification, biomarker discovery, or therapeutic prediction.

We excluded reviews, editorials, and studies that did not implement AI/ML methods.

## 3. Technologies Used in Microbiome Studies

Lately, AI has been a topical theme, emerging as one of the most advanced and powerful tools used to analyze any set or cluster of interrelated, connected, or dependent complex indeterminate information pertaining to the specific domain of microbiota. AI brings additional aspects of understanding the relationships between human health and gut microbiota. This is because of its capacity to process and analyze humongous amounts of data single handedly and also identify concealed correlations [27].

Table 1 shows the advancements in microbiome research that have led to the development of various technologies for microbial identification and functional analysis.

Each method has distinct advantages and challenges that impact its applicability in different research and clinical settings. Further, 16S rRNA profiling provides high sensitivity but has limitations in taxonomic resolution and functional characterization [35]. Reference-based metagenomics offers species- and strain-level classification and functional insights but depends heavily on the quality of reference databases [36]. MAGs facilitate species identification without amplification and contribute to expanding genomic catalogs, yet they struggle with complex datasets and repeat sequence assembly [37]. Multi-omic analysis, which integrates genomic, transcriptomic, proteomic, and metabolomic data, provides a comprehensive understanding of microbial functions but faces difficulties in data integration and temporal-spatial variability [38].

**Figure 1:**
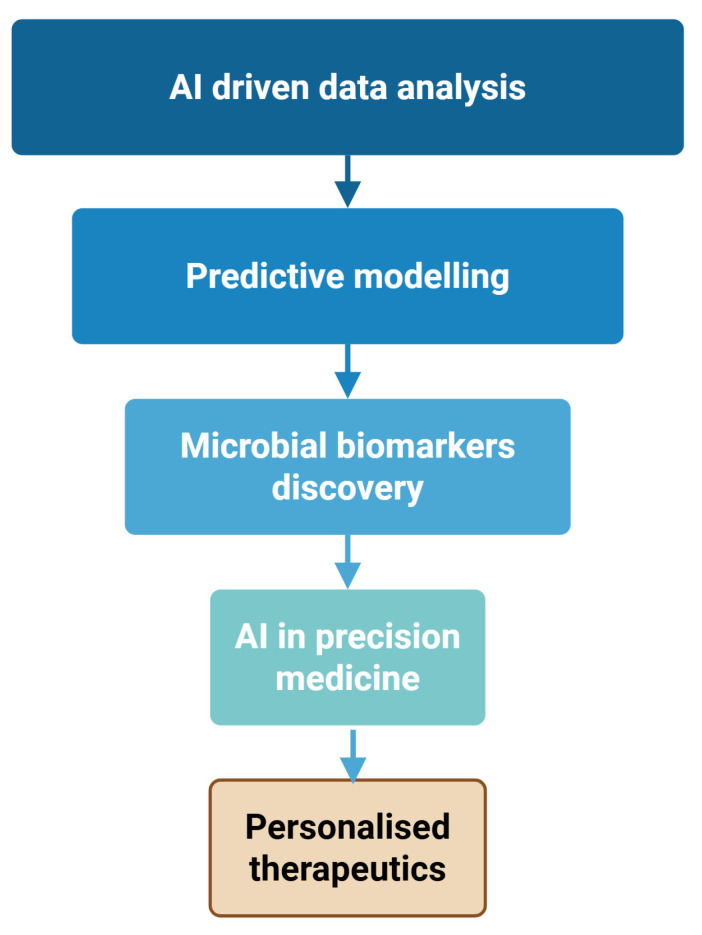
AI-Driven workflow for precision medicine in microbiome research. Created with Biorender [39].

Figure 1 shows that the recent developments in computer applications to analyze microbiome data enable researchers to complex bacterial datasets like never before.

Figure 1 demonstrates that the AI systems’ ability to recognize intricate patterns within massive amounts of data reveals critical relationships between microbial groups and human health.

AI technology, capable of analyzing large datasets to extract meaningful relationships, demonstrates remarkable correlations between groups of microbes and people’s health. Predictive models on how microbes interact are yet another aspect where AI is innovating [21]. These models explain the interactions of various microbial populations within the gastrointestinal tract, and their impacts on the metabolism and immunity of the host [40]. These findings are essential for epidemiology, as well as for developing precise therapies aimed at correcting the loss of keystone taxa. Searching for bacterial, biomarkers is one of the most advanced frontiers in medicine today [41]. AI can capture the distinctive microbial signatures associated with various health conditions. This is revolutionary for physicians as it enhances the diagnostic arsenal and facilitates the formulation of precise medical interventions [42]. The concept of targeted analysis of microbiota composition could profoundly affect dietary and lifestyle modifications based on a person’s specific microbial profile [43].

Machine learning, or ML, is a field of artificial intelligence that enables systems to learn and improve autonomously from data inputs. ML algorithms are mainly classified into unsupervised learning and supervised learning, which have been widely applied for analysis of gut microbiome [44]. Unsupervised learning methods overcome predefined dependent variables and discover hidden patterns within the provided datasets freely. This is why they are known as data-driven prediction. These algorithms can be divided into two groups, dimension reduction algorithms and clustering analysis algorithms [45]. Among the dimension reduction methods are principal components analysis (PCA), principal coordinate analysis, and t-distributed stochastic neighbor embedding (t-SNE) [46]. In omics data visualization, these methods are widely used to extract a set of principal variables from high-dimensional feature space [47]. Algorithms for clustering, which include partitioning or stratifying the set of objects into groups (clusters) based on similarities, often include k-means clustering, hierarchical clustering, and self-organizing map. Clustering analysis is particularly useful to identify novel patterns. They are applicable in the field of gut microbiota studies such as the discovery of human microbiota enterotypes and co-abundance gene groups [48].

**Figure 2:**
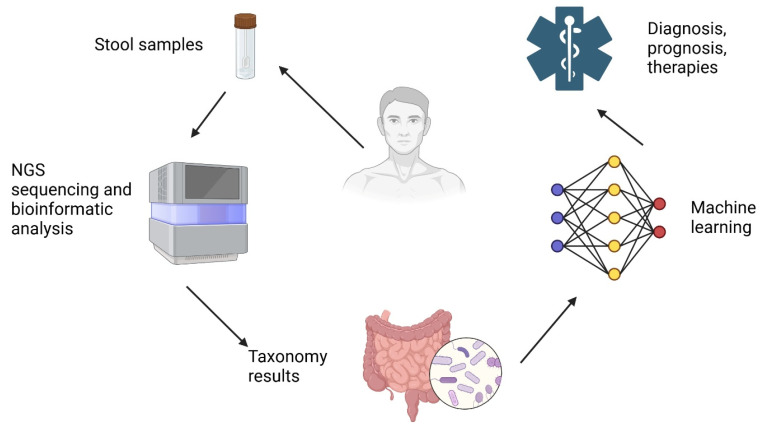
AI-driven microbiome analysis for precision medicine. Created with Biorender [30].

Figure 2 illustrates a workflow integrating next-generation sequencing (NGS) and machine learning for microbiome-based diagnostics and therapeutic applications.

The process begins with the collection of stool samples from patients, which are then subjected to NGS sequencing and bioinformatic analysis to identify microbial compositions [49]. The resulting taxonomy data provide insights into the gut microbiota, which is further analyzed using machine-learning algorithms. These AI-driven models process complex microbial patterns, enabling improved diagnosis, prognosis, and therapy recommendations for various health conditions.

### Investigating AI-Powered Techniques and Machine Learning Resources to Analyze the Intricate Microbiota

Table 2 highlights three key applications of AI in the field: predictive analysis, personalized probiotic recommendations, and clinical decision support.

For a clear comprehension of the linkages between the human diseases and the gut microbiota, characterization of the microbial communities and their features was done through next generation sequencing approaches such as amplicon sequencing and shotgun sequencing [57]. The most recent studies within the Metagenomics of the Human Intestinal Tract (MetaHIT) consortium and the Human Microbiome Project (HMP consisting of two phases HMP1 and HMP2), TEDDY study, have contributed for the enrichment of the human gut microbiota knowledge and the physiology consequences [58]. Thereafter, numerous sequence datasets of the human gut microbiome were made publicly available; for example, the Integrated Gene Catalog (IGC) [59], the Unified Human Gastrointestinal Genome (UHGG), and Protein (UHGP) catalogs with the astonishing number of 204,938 genomes of 4644 gut microbes and genes’ collections, which were identified [60]. Such powerful sequencing data allow for the assessment of the diversity of gut microbiome in various populations and to track changes in time [61,62]. Parallel to this newer high-throughput sequencing technologies are enabling new multi-omic approaches, which combine metabolomic, proteomic, genomic, and transcriptomic data from a variety of human tissues to portray a more comprehensive scope of human metabolism [63,64,65].

Recent advances have also introduced autoencoder-based dimensionality reduction techniques for microbiome analysis. Methods such as variational autoencoders (VAEs) and denoising autoencoders enable the compression of high-dimensional microbiome profiles into dense latent feature spaces while retaining biologically meaningful information [66].

## 4. Machine Learning

Machine learning plays a crucial role in modern healthcare, offering diverse approaches to data-driven decision-making [67].

Recent applications of machine learning in microbiome research illustrate how specific algorithms have been tailored to different study designs and data modalities. For instance, Random Forest classifiers have been widely employed to predict host phenotypes such as obesity, inflammatory bowel disease, and colorectal cancer using 16S rRNA sequencing data, achieving robust performance in distinguishing case–control cohorts [68]. Similarly, Support Vector Machines have demonstrated strong discriminatory power in shotgun metagenomic datasets, particularly in disease classification tasks where subtle microbial compositional changes must be detected [69]. More recently, deep-learning architectures, including convolutional neural networks, have been applied to high-dimensional multi-omics datasets for biomarker discovery, enabling the identification of non-linear associations between microbial genes, metabolites, and host clinical parameters [70].

Table 3 shows that supervised learning, which relies on labelled datasets, is widely used for disease diagnosis, drug discovery, and personalized medicine through classification and regression models.

In order to better illustrate their applicability, each machine-learning category can be contextualized with examples from microbiome research. In supervised learning, Random Forest classifiers have been widely used to distinguish colorectal cancer patients from healthy controls based on metagenomic profiles, demonstrating strong predictive performance in disease classification tasks [81]. Unsupervised learning approaches, particularly clustering methods, have uncovered distinct gut enterotypes across human populations, providing insights into microbial community structure and its links to diet and host metabolism [82].

On the other hand, unsupervised learning detects hidden patterns within data, making it valuable for clustering similar disease profiles and identifying new biomarkers [83]. Semi-supervised learning combines both labelled and unlabelled data to improve model accuracy, aiding in medical imaging analysis and clinical trial predictions [84]. Lastly, reinforcement learning operates through an agent-environment interaction, optimizing treatment strategies and understanding drug-microbiota interactions. Each ML type has unique advantages. Selecting the right approach depends on the specific healthcare application and data availability [85].

**Figure 3:**
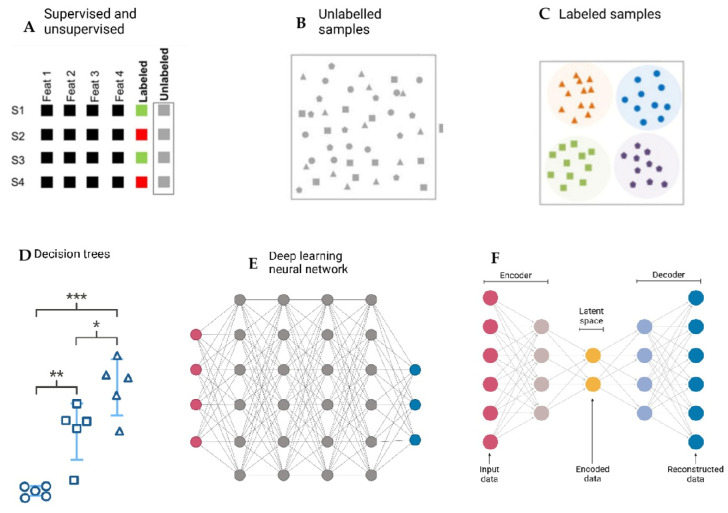
Overview of Machine-Learning approaches in data classification and decision-making. (**A**). Supervised learning: algorithms trained on labeled data to predict outcomes (e.g., Random Forest, SVM). (**B**). Unsupervised learning: algorithms detecting hidden patterns and clusters in unlabeled data (e.g., k-means clustering). (**C**). Semi-supervised learning: hybrid models combining small labeled datasets with large unlabeled datasets. (**D**). Reinforcement learning: agent–environment interactions optimized through rewards and penalties (*→First-level decision split; ** → Second-level decision split; *** → Third-level decision split; Circle: Class/category 1; Square: Class/category 2; Triangle: Class/category 3). (**E**). Deep-learning architectures: neural networks with multiple layers, useful for complex pattern recognition in high-dimensional data. (**F**). Dimensionality reduction/feature extraction: techniques such as PCA or autoencoders used to compress high-dimensional microbiome datasets into interpretable forms. Created with Biorender [39].

Figure 3 provides a visual representation of different machine-learning approaches used for data classification and decision-making.

It can be seen that supervised learning relies on labeled datasets to train algorithms, while unsupervised learning identifies patterns in unlabeled data. Also, different machine-learning models include decision trees, deep-learning neural networks, and autoencoders. Decision trees illustrate hierarchical decision-making based on input features, whereas deep-learning neural networks involve multiple interconnected layers that process complex patterns [86]. The autoencoder model, consisting of an encoder and decoder, reduces dimensionality by encoding input data into a latent space before reconstructing it [87].

To address the translational value of machine learning in microbiome studies, we expanded our discussion beyond theoretical descriptions. In Table 4, we summarize representative published applications of AI/ML in gut microbiome research over time. We specified the algorithm used, the type of dataset, and the study outcome.

Random Forest algorithms have been successfully used for colorectal cancer classification [88,91], while Support Vector Machines were applied in early work on type 2 diabetes prediction [89]. Clustering techniques enabled the discovery of distinct gut enterotypes across populations [90]. More recently, deep-learning approaches, including convolutional neural networks, have been utilized for biomarker discovery and improved disease prediction [92,94]. Logistic regression and Random forest methods were also applied to 16S rRNA datasets to predict host phenotypes and microbial dynamics [93]. Furthermore, reinforcement learning frameworks have been explored to optimize diet–microbiome interactions in the context of cardiovascular risk [95].

To move beyond general statements, in Table 5 we report key quantitative outcomes from representative microbiome AI/ML studies.

In colorectal cancer, Zeller et al. [88] trained metagenomic classifiers and showed that stool–metagenome detection achieved accuracy comparable to the FOBT; importantly, combining metagenomics with FOBT increased sensitivity by > 45% at matched specificity, and performance was validated across independent cohorts (N = 335) from multiple countries. In their multi-study meta-analysis of 768 fecal metagenomes, Wirbel et al. [91] identified a core set of 29 CRC-enriched species and demonstrated that models trained on multiple studies generalized better across cohorts than single-study models, with validation in three independent populations. For type 2 diabetes, Qin et al. [89] performed a two-stage MGWAS (N = 345) and reported that the derived microbial gene markers supported classification of T2D in an additional validation set, establishing an early predictive use case for metagenomic features. Extending to European women (N = 145), Karlsson et al. [96] built a metagenome-based classifier that identified T2D with high accuracy and flagged individuals with impaired glucose tolerance as “diabetes-like”. At the benchmarking level, Pasolli et al. [92] analyzed 2424 metagenomes across eight studies and reported cross-validated AUCs that quantify the discriminative ceiling achievable from shotgun data: liver cirrhosis AUC = 0.945 (95% CI 0.909–0.981), CRC AUC = 0.873 (0.802–0.944), and IBD AUC = 0.890 (0.812–0.968).

Complementing these results on 16S rRNA data, Topçuoğlu et al. [93] benchmarked seven models for detecting colonic screen-relevant neoplasia (SRNs; n = 490) and found Random Forest AUROC = 0.695 (IQR 0.651–0.739), with L2-regularized logistic regression AUROC = 0.680 (IQR 0.625–0.735), quantifying realistic performance and the small gap between complex and interpretable models under rigorous evaluation.

Beyond disease labels, unsupervised structure has also been quantified. Arumugam et al. [90] identified three robust enterotypes from multi-country metagenomes and confirmed them in larger cohorts, demonstrating stratified, not continuous, organization of gut communities that underpins downstream predictive modeling. For host/environment prediction from 16S, the MicroPheno approach (k-mer features with ML/deep learning) achieved macro-F1 = 0.88 (18 ecological environments) and 0.87 (five organismal environments), and outperformed OTU-based baselines; deep networks surpassed classical ML on larger datasets.

To illustrate model performance with concrete metrics, we also summarize two other representative, large-scale T2D microbiome prediction studies. First, Reitmeier et al. [97] trained machine-learning models on a German population cohort (KORA; n = 1976) using an arrhythmic bacterial signature derived from time-stamped stool profiles. Cross-validated classifiers distinguished T2D from non-T2D with an AUC of 0.73, which improved to 0.79 when combined with BMI; external validation achieved AUC = 0.76 in an independent cohort (FoCus; n = 1363), and prospective prediction in 699 KORA participants 5 years later reached AUC = 0.78 (all using the same arrhythmic taxa panel). Second, in a large cross-sectional study in China (n = 1160; metagenomics on a well-phenotyped subset), integrating plasma choline with specific microbial species and standard risk factors yielded an AUC of 0.971 for classifying diabetes versus controls, highlighting the gain from combining host metabolites with microbiome features [98].

An important aspect of applying machine learning to microbiome datasets is the use of appropriate preprocessing strategies, as recommended by initiatives such as ML4Microbiome [99]. Since microbiome data are inherently compositional, transformations such as the centered log-ratio or additive log-ratio are commonly applied to account for relative abundance constraints [100]. Additionally, filtering of rare taxa helps reduce noise and improve model stability, while normalization techniques address sparsity and uneven sequencing depth across samples. Beyond preprocessing, several workflows have emerged as best practices for robust model development and interpretation. For example, the combination of Statistically Equivalent Signatures with Random Forest provides reliable feature selection, while logistic regression coupled with Individual Conditional Expectation plots enables interpretable predictions and facilitates the identification of microbial signatures most relevant to clinical outcomes [99].

Another important challenge in microbiome-based machine learning is the presence of systematic biases in compositional data. For example, reference genome incompleteness can limit accurate taxonomic assignment, leaving many microbial reads unclassified or misclassified [101]. In addition, horizontal gene transfer complicates functional inference, since genes with key metabolic roles may not be unique to specific taxa, blurring associations between microbial identity and function [102]. Further, annotation inconsistencies across different reference databases reduce reproducibility and hinder cross-study comparability. To mitigate these issues, recent efforts have focused on creating harmonized and comprehensive reference catalogs such as the Unified Human Gastrointestinal Genome [103] and the Integrated Gene Catalog [104], which expand genomic coverage and improve the consistency of downstream analyses.

## 5. Ethical, Legal, and Social Implications

The integration of AI into microbiome research also raises important ethical, legal, and social considerations that must be addressed to ensure responsible translation into clinical practice. One key concern is algorithmic bias, which can arise when models are trained on datasets that underrepresent certain populations, leading to reduced accuracy and inequitable outcomes in minority groups [105]. Issues of data access and equity further complicate this landscape, as not all institutions or regions have equal resources to generate and share high-quality microbiome datasets. Additionally, privacy concerns related to the storage and use of genomic and health-associated data highlight the need for secure frameworks that safeguard sensitive information [106]. Finally, there are significant regulatory challenges, particularly in defining standards and guidelines for the approval and clinical adoption of AI-driven microbiome tools.

## 6. Existing Challenges and Future Directions for AI in Microbiome-Based Healthcare

Although artificial intelligence is gaining attention in microbiome-related disease research, few models have made it to clinical practice, which can be assumed to stem from the lack of robustness and generalizability [107]. This is an issue coming from attempts to validate a model, and this sometimes goes wrong in data processing and training [108].

Another significant challenge for the translational scientific investigations is the AI model’s typical opacity. In situations wherein an AI model meets business requirements, determining the logic behind its action is exceedingly complex. While random forests and similar techniques can analyze the relevance of a feature for the model’s decision, other forms of machine learning have to be evaluated with regard to the significance of features separately [109].

Another common problem is data leakage, which occurs if data are not sufficiently divided into training and test subsets, resulting in method-learning shortcuts, which culminates in over reporting and poor performance in practice [60].

An essential component in training sophisticated AI models is the availability of thorough, substantial, and high-quality microbiome data. There have been several failed international attempts in the past for microbiome data collection, such as the Human Microbiome Project and the MetaHIT project (as previously mentioned), which have now opened new endeavors like the Million Microbiomes from Humans Project [58].

## 7. Conclusions

The massive volumes of gut metagenomic data available now allow ML technologies to be applied for the identification of many uncharacterized microbial genomes and proteins, something which was previously impossible. This represents a first step towards a deeper mechanistic understanding of the gut microbiome. Moreover, these unexplogenic protein sequences can be further analyzed through ML to predict protein structure and aid in enzyme or drug design. Additionally, ML can aid in the development of probiotics, as well as in the creation of synthetic consortia of multiple species of microbes. Hence, these ML approaches will eventually facilitate achieving personalized nutrition and targeted medicine at the microbiome level.

However, alongside these opportunities, several challenges and limitations must be recognized. A major concern remains data standardization, as microbiome studies often employ heterogeneous sequencing platforms, pipelines, and reference databases that complicate reproducibility and cross-study comparisons. Another critical issue is clinical applicability: while many AI-driven models demonstrate high performance in research settings, their translation into clinical practice requires robust validation in large, diverse cohorts, and the establishment of regulatory pathways.

A critical concern is the risk of data leakage when training and test sets are not strictly separated, which can artificially inflate model performance; to avoid this, nested cross-validation, validation on independent external cohorts, and multi-population benchmarking are recommended as best practices.

Furthermore, ethical considerations, including data privacy, patient consent, and algorithmic bias, must be carefully addressed to ensure responsible and equitable implementation of AI in healthcare.

There are countless challenges, but the arrival of AI and big data analytics has opened doors to many applications of the gut microbiota, creating immense opportunities for developing novel strategies aimed at disease treatment and prevention through modification of the gut microbiota.

## Figures and Tables

**Table 1:** Comparative analysis of microbiome profiling technologies: Advantages and limitations.

Technology	Benefits	Limitations
16S rRNA profiling [28]	- Offers high sensitivity for microbial identification	- Limited taxonomic resolution - Susceptible to PCR amplification biases - Functional insights rely on indirect extrapolation
Reference-based metagenomics [29]	- Enables species- and strain-level classification for certain taxa - Integrates functional data - Does not require amplification	- Strongly influenced by the quality and diversity of reference databases - Unable to distinguish between expressed and non-expressed genes
Metagenome-assembled genomes (MAGs) [30]	- No need for amplification - Identifies species and evolutionary relationships within a sample - Helps expand reference databases	- Challenges in handling complex datasets - Difficulties in assembling repetitive sequences
Multi-omic analysis [31]	- Detects functional genes, RNA transcripts, proteins, and metabolites - Provides insights into potential biological mechanisms	- Complex data integration - Issues related to temporal and spatial variations in sampling
ITS sequencing (Internal Transcribed Spacer) [32]	High resolution for fungal community profiling; complementary to 16S rRNA for bacterial communities	Limited to fungal identification; subject to PCR biases; less standardized databases
Microarrays (PhyloChip, GeoChip) [33]	High-throughput; detects thousands of taxa/functional genes simultaneously; reproducible results	Limited to predefined probes; cannot detect novel organisms; requires robust probe design
FISH (Fluorescence In Situ Hybridization) [34]	Visualizes microbes in situ; provides spatial organization; no need for cultivation	Lower sensitivity for rare taxa; requires specific probes; limited multiplexing

**Table 2:** Detailed overview of how AI is shaping probiotic applications in clinical settings.

Category	Description	Function	What it Can Be Used?
Predictive analysis [50]	AI forecasts how various probiotic strains interact with the gut microbiome and their potential effects on health, using past clinical trials and datasets [51]	It can determine the most effective probiotic strains for specific health conditions or demographic groups.	An AI model may predict that a specific combination of *Lactobacillus* and *Bifidobacterium* strains effectively alleviates IBS symptoms in adults [52].
Customized probiotic suggestions [53]	AI enables tailored probiotic recommendations based on an individual’s microbiome composition and overall health status.	AI designs personalized probiotic formulations suited for each patient using genomic, metabolomic, and clinical information.	AI proposes a specialized probiotic formula to restore microbial equilibrium for individuals with an imbalanced gut microbiome and metabolic issues.
AI-assisted clinical guidance [54]	AI helps healthcare providers choose appropriate probiotic therapies by analyzing patient records and predicting treatment outcomes.	AI-driven systems offer data-supported recommendations for probiotic interventions, aiding in chronic disease management or post-antibiotic recovery.	AI assesses patient data to recommend probiotics that reduce the risk of antibiotic-associated diarrhea and enhance gut health.
AI-driven probiotic strain discovery [55]	Machine-learning algorithms identify novel probiotic strains with desirable metabolic or immunomodulatory traits.	Screens microbial genomes and predicts beneficial functionalities.	AI discovers new probiotic candidates with anti-inflammatory or cholesterol-lowering properties for targeted therapies.
Clinical trial simulation & validation [56]	AI models simulate probiotic interventions and predict trial outcomes before or alongside human studies.	Reduces trial costs and accelerates validation of probiotic efficacy.	AI predicts probiotic success rates in T2D management or validates formulations in silico before moving to clinical phases.

**Table 3:** Types of Machine Learning and their applications in healthcare.

ML Type	Description	Labeled Data?	Output Type	Common Algorithms	Where it Can Be Used?
Supervised learning [71,72,73]	Models are trained on labeled datasets with known inputs and outputs to make predictions.	Yes (fully labeled)	Classification labels, regression values	Linear/Logistic Regression, Decision Trees, Random Forests, Support Vector Machines (SVM), Gradient Boosting	Disease diagnosis (e.g., cancer classification from microbiome), biomarker discovery, clinical outcome prediction, medical imaging analysis
Unsupervised learning [74,75]	Finds hidden structures or patterns in unlabeled data.	No	Clusters, latent structures, reduced feature spaces	K-means clustering, Hierarchical clustering, Self-Organizing Maps, Neural Networks, Dimensionality reduction (PCA, ICA, t-SNE)	Identification of gut enterotypes, discovery of microbial community patterns, patient stratification for personalized treatment
Semi-supervised learning [76,77,78]	Uses both labeled and unlabeled data to improve performance.	Yes (partially labeled)	Improved predictive models leveraging partial labels	Self-training, Co-training, Graph-based methods	Rare disease classification, clinical trial predictions with incomplete data, microbiome classification when full annotations are missing
Reinforcement learning [79,80]	An agent learns by interacting with the environment and receiving feedback (rewards/penalties).	No	Policies, reward signals	Q-learning, Deep Reinforcement Learning	Optimizing personalized treatment plans, adaptive probiotic recommendations, drug–microbiome interaction modeling

**Table 4:** Applications of AI/ML in gut microbiome research.

Study	Algorithm	Data Type	Application/Outcome
Zeller et al. [88]	Random forest	Metagenomic sequencing	Disease classification: identified microbial signatures distinguishing colorectal cancer from controls
Qin et al. [89]	Support Vector machines	Metagenomic	Type 2 diabetes classification and biomarker discovery from gut microbiome profiles
Arumugam et al. [90]	Clustering/Unsupervised learning	Metagenomic	Discovery of human gut enterotypes across populations
Wirbel et al. [91]	Random forest, LASSO regression	Metagenomic	Developed multi-cohort model for colorectal cancer prediction and validated across external cohorts
Pasolli et al. [92]	Deep-learning neural networks	Shotgun metagenomic	Improved disease classification (IBD, obesity, CRC) across large-scale microbiome datasets
Topçuoğlu et al. [93]	Logistic regression, Random forest	16S rRNA amplicon	Predictive modeling of host phenotypes and experimental outcomes from microbiome data
Asgari et al. [94]	Convolutional neural networks	Metagenomic	Biomarker discovery for host–microbiome associations and disease prediction
Vilne et al. [95]	Reinforcement learning models	Diet–microbiome interaction data	Optimized microbiome-based risk prediction models for coronary artery disease

**Table 5:** Quantitative outcomes from representative microbiome AI/ML studies.

Study	Sample Size (N)	Data Type	Algorithm(s)	Validation	Performance Metric(s)
Zeller et al. [88]	335 (France, Germany, Italy, Austria)	Metagenomic	Random forest	Cross-cohort	Metagenomics + fecal occult blood test (FOBT) sensitivity increased >45% vs. FOBT alone at matched specificity
Wirbel et al. [91]	768 metagenomes, 3 validation cohorts	Metagenomic	Random forest, LASSO	Multi-study CV + external validation	Cross-study AUCs stable with multi-study training; identified 29 robust CRC-associated species
Qin et al. [89]	345 (Chinese adults)	Metagenomic	SVM	Train/validation split	Gene markers classified T2D vs. controls with significant separation
Karlsson et al. [96]	145 (European women)	Metagenomic	SVM, logistic regression	Internal CV	High accuracy for T2D vs. controls; identified pre-diabetic individuals as “diabetes-like”
Pasolli et al. [92]	2424 metagenomes across 8 studies	Shotgun metagenomic	Deep learning, Random forest, SVM	Cross-validation + cross-study	Cirrhosis AUC = 0.945; CRC AUC = 0.873; IBD AUC = 0.890
Topçuoğlu et al. [93]	490 stool 16S rRNA	RF, logistic regression, others	Repeated CV	RF AUROC = 0.695 (IQR 0.651–0.739); Logistic regression AUROC = 0.680 (0.625–0.735)	
Arumugam et al. [90]	Multi-country cohorts	Metagenomic	Clustering (k-means, PCA)	Replication across cohorts	Identified 3 reproducible enterotypes, stable across populations
Asgari et al. [94]	16S datasets, multiple environments	Deep learning (MicroPheno)	Cross-validation	Macro-F1 = 0.88 (18 environments); 0.87 (5 organismal environments)	
Vilne et al. [95]	Diet + metagenomic cohort (CAD study)	Multi-omic	Reinforcement learning	Case study analysis	Optimized diet–microbiome interactions for CAD risk prediction

## Data Availability

No new data were created.
